# Crystal structure of 2,3-bis­(4-methyl­phen­yl)benzo[*g*]quinoxaline

**DOI:** 10.1107/S2056989018004413

**Published:** 2018-03-23

**Authors:** Young-Inn Kim, Seong-Jae Yun, Sung Kwon Kang

**Affiliations:** a Department of Chemistry Education and Department of Chemical Materials, Graduate School, Pusan National University, Busan 46241, South Korea; b Department of Chemistry, Chungnam National University, Daejeon 34134, South Korea

**Keywords:** crystal structure, 2,3-di-*p*-tolyl­benzo[*g*]quinoxaline, N-heterocyclic compound, C—H⋯π inter­action

## Abstract

The title compound, C_26_H_20_N_2_, was obtained during a search for new π-extended ligands with the potential to generate efficient phosphors with iridium(III) for organic light-emitting devices (OLEDs). The benzoquinoxaline ring system is almost planar (r.m.s. deviation = 0.076 Å). A pseudo-twofold rotation axis runs through the midpoints of the C2—C3 and C9—C10 bonds. The two phenyl rings are twisted relative to the benzoquinoxaline ring system, making dihedral angles of 53.91 (4) and 36.86 (6)°. In the crystal, C—H⋯π (arene) inter­actions link the mol­ecules, but no π–π inter­actions between aromatic rings are observed.

## Abstract 2

The synthesis and crystal structure of 2,3-di-*p*-tolyl­benzo[*g*]quinoxaline, a potential ligand for OLED Ir^III^ complexes, are reported.

## Chemical context   

Quinoxalines are well-known nitro­gen-containing heterocyclic compounds, and substituted quinoxalines are important ligands with transition metals (Achelle *et al.*, 2013 ▸; Floris *et al.*, 2017 ▸; Tariq *et al.*, 2018 ▸). They act as chelating agents bearing ring complexes bounded by a benzene ring and a pyrazine ring. We have reported, for example, deep-red emissive iridium(III) complexes containing 2,3-di­phenyl­quinoxaline (dpqH), in which red emissions contributed to the conjugated structure of the dpq ligand (Song *et al.*, 2015 ▸). The use of long conjugated compounds as metal coordination ligands could be an approach to develop novel emitters toward red-shift emission up to near-infrared (NIR) wavelengths due to inter­system crossing (Ahn *et al.*, 2009 ▸). Recently, 2,3-di­phenyl­benzoquinoxaline (dpbqH), a more π-extended ligand than dpqH, has been introduced, and its iridium(III) complex showed bathochromic shifted emission at 763 nm (Kim *et al.*, 2018 ▸). The aromatic rings in dpqH formed dimeric aggregates by π–π inter­actions, and these dimers inter­act *via* van der Waals inter­actions in the solid state (Cantalupo *et al.*, 2006 ▸; Kim *et al.*, 2018 ▸). In this work, we have synthesized 2,3-di-*p*-tolyl­benzo[*g*]quinoxaline (dmpbqH) from the reaction of 4,4-di­methyl­benzil with 2,3-di­aminona­phthalene, and investigated its single crystal structure.
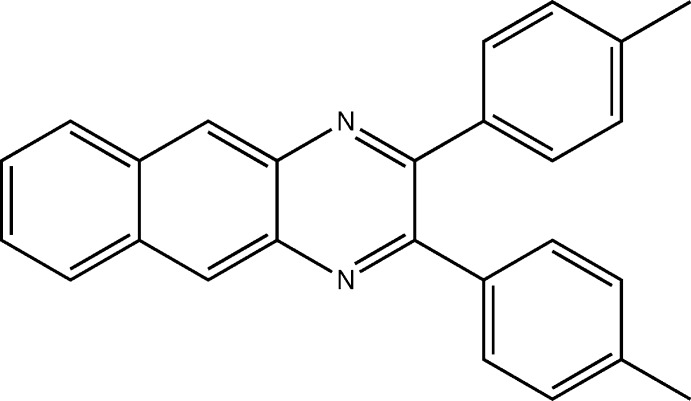


## Structural commentary   

**Figure 1:**
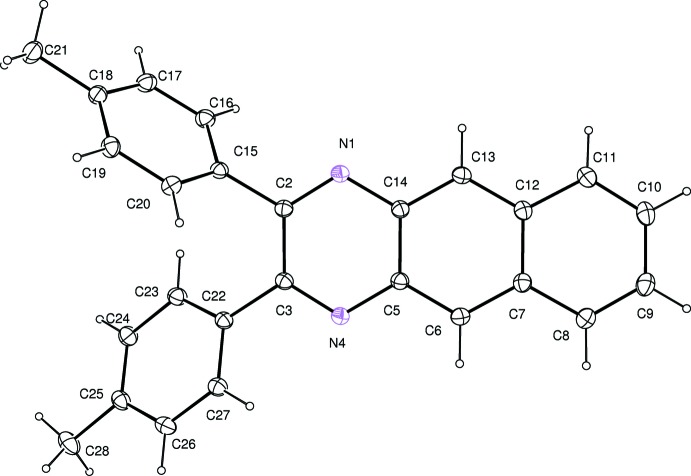
Mol­ecular structure of the title compound, showing the atom-numbering scheme and 30% probability ellipsoids for non-H atoms.

The mol­ecular structure of the title compound is shown in Fig. 1 ▸. The benzoquinoxaline ring system (atoms N1/C2/C3/N4/C5–C14) is almost planar, with an r.m.s. deviation of 0.076 Å from the corresponding least-squares plane defined by the 14 constituent atoms. In the pyrazine heterocyclic ring, the N1—C2 [1.310 (2) Å] and C3—N4 [1.310 (2) Å] bonds are shorter than the N1—C14 [1.381 (2) Å] and N4—C5 [1.379 (2) Å] bonds, even though the pyrazine ring has a delocalized π-system. There is a pseudo-twofold rotation axis passing through the midpoints of the C2—C3, C5—C14, C7—C12, and C9—C10 bonds. The two phenyl rings (atoms C15–C20 and C22–C25) are twisted relative to the benzoquinoxaline ring system, making dihedral angles of 53.91 (4) and 36.86 (6)°, respectively. The dihedral angle between the phenyl rings is 65.22 (6)°.

## Supra­molecular features   

**Figure 2:**
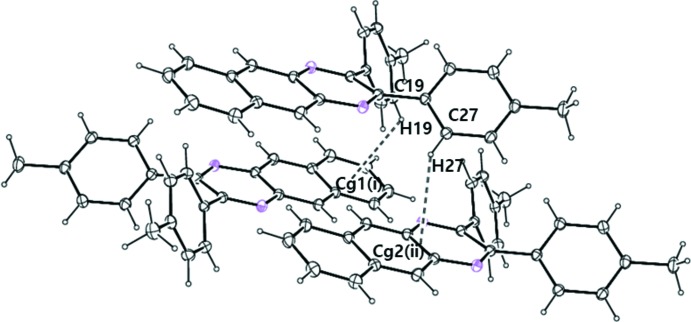
Part of the crystal packing showing mol­ecules linked by inter­molecular C—H⋯π inter­actions (Table 1 ▸; shown as dashed lines). *Cg*1 and *Cg*2 are the centroids of the C7–C12 and the N1/C2/C3/N4/C5–C7/C12–C14 rings, respectively. [Symmetry codes: (i) −*x* + 1, −*y*, −*z*, (ii) *x* − 1, *y*, *z*].

**Figure 3:**
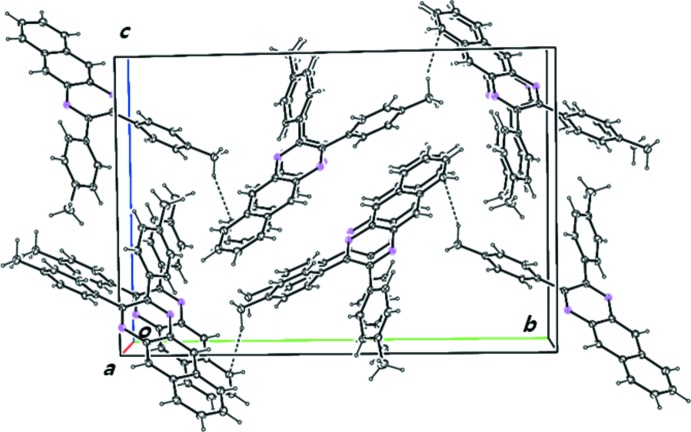
Crystal packing of the title compound, showing mol­ecules linked by inter­molecular C—H⋯π bonds (dashed lines).

In the crystal, there are two C—H⋯π inter­actions: C19—H19⋯*Cg*1^i^ and C27—H27⋯*Cg*2^ii^ (Table 1 ▸, Fig. 2 ▸) which stabilize the crystal packing (Fig. 3 ▸). There are no π–π inter­actions between the aromatic rings.

## Database survey   

A search of the Cambridge Structural Database (CSD; Groom *et al.*, 2016 ▸) *via* the WebCSD inter­face in February 2018 returned several entries for crystal structures related to 2,3-disubstituted benzoquinoxalines. In 2,3-di­phenyl­benzoquinoxaline, the two phenyl rings form dihedral angles of 43.42 (3) and 46.89 (3)° with the benzoquinoxaline plane, a little larger than those of the title compound. The packing in the crystals is described as having a herringbone motif (REKDIV, Cantalupo *et al.*, 2006 ▸; REKDIV01, Chan & Chang, 2016 ▸). There are three entries for metal complexes with this ligand. In the crystal lattice of a bis-cyclo­manganese complex, the mol­ecules are π-stacked in a parallel head-to-tail pattern with a mean inter-planar distance between the benzoquinoxaline planes of 3.5 Å (DECTAH; Djukic *et al.*, 2005 ▸). In addition we also found two octa­hedral Ir^III^ complexes (VEHCAN and VEHCER; Chen *et al.*, 2006 ▸).

There are three entries for crystal structures related to 2,3-bis­(2-pyrid­yl)benzoquinoxaline. In the distorted octa­hedral Co^III^ complex (JUHVIR; Escuer *et al.*, 1991 ▸), the Co^III^ atom is situated in the benzoquinoxaline plane, coordinated by one pyridyl N atom and one quinoxaline N atom. In the octa­hedral Re^V^ complex (HAYSAB; Bandoli *et al.*, 1994 ▸), the Re^V^ atom is chelated by two pyridyl N atoms of the bis­(2-pyrid­yl)benzoquinoxaline ligand. Finally, in the square-planar Pt^II^ complex (AYAMIW; Cusumano *et al.*, 2004 ▸), the benzo­quin­oxaline moiety lies almost perpendicular to the square plane giving the mol­ecule an unusual L-shaped geometry.

## Synthesis and crystallization   

Chemicals were obtained commercially in reagent grade and used as received. Solvents were dried using standard procedures as described in the literature. ^1^H NMR spectra were recorded with a 300 MHz Varian Mercury model in CDCl_3_. 4,4-Di­methyl­benzil (3 mmol), 2,3-di­aminona­phthalene (4.4 mmol), and iodine (0.37 mmol) were dissolved slowly in aceto­nitrile (10 ml), and stirred for 10 minutes at room temperature. The reaction mixture was poured into water, extracted with ether and dried over anhydrous MgSO_4_. After volatiles had been removed under reduced pressure, the product was purified by silica gel chromatography using an eluent of hexa­ne/ethyl acetate (20:1). Pale-yellow single crystals of the title compound were obtained from di­chloro­methane/hexane (1:1) solution within a few days by slow evaporation of the solvent at 298 K, yield: 48%. ^1^H NMR (300 MHz, CDCl_3_): 8.72 (*s*, 2H), 8.12 (*m*, 2H), 7.56 (*m*, 2H), 7.49 (*d*, 2H, *J* = 8.1Hz), 7.18 (*d*, 2H, *J* = 7.8Hz), 2.40 (*s*, 6H).

## Refinement   

Crystal data, data collection and structure refinement details are summarized in Table 2 ▸. The C-bound H atoms were positioned geometrically and refined using a riding model, with *d*(C—H) = 0.93–0.96 Å, and with *U*_iso_(H) = 1.2*U*_eq_(C) for aromatic-H and 1.5*U*_eq_(C) for methyl-H atoms, respectively.

## Supplementary Material

Crystal structure: contains datablock(s) I. DOI: 10.1107/S2056989018004413/vm2210sup1.cif

Structure factors: contains datablock(s) I. DOI: 10.1107/S2056989018004413/vm2210Isup2.hkl

Supporting information file. DOI: 10.1107/S2056989018004413/vm2210Isup3.cml

CCDC reference: 1543571

Additional supporting information:  crystallographic information; 3D view; checkCIF report

## Figures and Tables

**Table 1:** Hydrogen-bond geometry (Å, °) *Cg*1 and *Cg*2 are the centroids of atoms C7–C12 and N1/C2/C3/N4/C5–C7/C12–C14, respectively.

*D*—H⋯*A*	*D*—H	H⋯*A*	*D*⋯*A*	*D*—H⋯*A*
C19—H19⋯*Cg*1^i^	0.93	2.88	3.488 (3)	124
C27—H27⋯*Cg*2^ii^	0.93	2.91	3.601 (3)	132

Symmetry codes: (i) 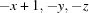; (ii) 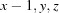.

**Table 2:** Experimental details

Crystal data	
Chemical formula	C_26_H_20_N_2_
*M*_r_	360.44
Crystal system, space group	Monoclinic, *P*2_1_/*n*
Temperature (K)	296
*a*, *b*, *c* (Å)	6.0814 (1), 21.5212 (4), 14.8312 (3)
β (°)	91.2496 (11)
*V* (Å^3^)	1940.63 (6)
*Z*	4
Radiation type	Mo *K*α
μ (mm^−1^)	0.07
Crystal size (mm)	0.15 × 0.12 × 0.10

Data collection	
Diffractometer	Bruker SMART CCD area-detector
No. of measured, independent and observed [*I* > 2σ(*I*)] reflections	29811, 4805, 2628
*R*_int_	0.050
(sin θ/λ)_max_ (Å^−1^)	0.667

Refinement	
*R*[*F* ^2^ > 2σ(*F* ^2^)], *wR*(*F* ^2^), *S*	0.049, 0.124, 1.01
No. of reflections	4805
No. of parameters	255
H-atom treatment	H-atom parameters constrained
Δρ_max_, Δρ_min_ (e Å^−3^)	0.17, −0.16

Computer programs: *SMART* and *SAINT* (Bruker, 2012 ▸), *SHELXS2013* (Sheldrick, 2008 ▸), *SHELXL2014* (Sheldrick, 2015 ▸) and *ORTEP-3 for Windows* and *WinGX* (Farrugia, 2012 ▸).

## supplementary crystallographic information

### Crystal data

**Table:**

C_26_H_20_N_2_	*F*(000) = 760
*M**_r_* = 360.44	*D*_x_ = 1.234 Mg m^−^^3^
Monoclinic, *P*2_1_/*n*	Mo *K*α radiation, λ = 0.71073 Å
*a* = 6.0814 (1) Å	Cell parameters from 3900 reflections
*b* = 21.5212 (4) Å	θ = 2.3–21.9°
*c* = 14.8312 (3) Å	µ = 0.07 mm^−^^1^
β = 91.2496 (11)°	*T* = 296 K
*V* = 1940.63 (6) Å^3^	Block, yellow
*Z* = 4	0.15 × 0.12 × 0.10 mm

### Data collection

**Table:**

Bruker SMART CCD area-detector diffractometer	*R*_int_ = 0.050
Radiation source: fine-focus sealed tube	θ_max_ = 28.3°, θ_min_ = 1.7°
φ and ω scans	*h* = −8→8
29811 measured reflections	*k* = −26→28
4805 independent reflections	*l* = −19→19
2628 reflections with *I* > 2σ(*I*)	

### Refinement

**Table:**

Refinement on *F*^2^	0 restraints
Least-squares matrix: full	Hydrogen site location: inferred from neighbouring sites
*R*[*F*^2^ > 2σ(*F*^2^)] = 0.049	H-atom parameters constrained
*wR*(*F*^2^) = 0.124	*w* = 1/[σ^2^(*F*_o_^2^) + (0.0451*P*)^2^ + 0.2858*P*] where *P* = (*F*_o_^2^ + 2*F*_c_^2^)/3
*S* = 1.00	(Δ/σ)_max_ < 0.001
4805 reflections	Δρ_max_ = 0.17 e Å^−^^3^
255 parameters	Δρ_min_ = −0.16 e Å^−^^3^

### Special details

**Table:**

Geometry. All esds (except the esd in the dihedral angle between two l.s. planes) are estimated using the full covariance matrix. The cell esds are taken into account individually in the estimation of esds in distances, angles and torsion angles; correlations between esds in cell parameters are only used when they are defined by crystal symmetry. An approximate (isotropic) treatment of cell esds is used for estimating esds involving l.s. planes.

### Fractional atomic coordinates and isotropic or equivalent isotropic displacement parameters (Å^2^)

**Table:**

	*x*	*y*	*z*	*U*_iso_*/*U*_eq_	
N1	0.7331 (2)	0.02999 (6)	0.12787 (9)	0.0539 (4)	
C2	0.5746 (3)	0.02628 (7)	0.18606 (11)	0.0496 (4)	
C3	0.4315 (3)	0.07859 (8)	0.20406 (11)	0.0496 (4)	
N4	0.4398 (2)	0.12958 (7)	0.15602 (9)	0.0551 (4)	
C5	0.5980 (3)	0.13326 (8)	0.09102 (11)	0.0513 (4)	
C6	0.6102 (3)	0.18621 (8)	0.03848 (12)	0.0595 (5)	
H6	0.5033	0.2169	0.0436	0.071*	
C7	0.7797 (3)	0.19434 (8)	−0.02186 (11)	0.0549 (4)	
C8	0.7963 (3)	0.24860 (9)	−0.07635 (13)	0.0683 (5)	
H8	0.6866	0.2787	−0.0744	0.082*	
C9	0.9686 (3)	0.25701 (10)	−0.13079 (13)	0.0728 (6)	
H9	0.9765	0.2926	−0.1661	0.087*	
C10	1.1356 (3)	0.21237 (10)	−0.13438 (13)	0.0728 (6)	
H10	1.2561	0.2195	−0.1706	0.087*	
C11	1.1252 (3)	0.15891 (9)	−0.08612 (12)	0.0647 (5)	
H11	1.2362	0.1294	−0.0908	0.078*	
C12	0.9451 (3)	0.14766 (8)	−0.02829 (11)	0.0529 (4)	
C13	0.9257 (3)	0.09318 (8)	0.02196 (11)	0.0558 (4)	
H13	1.0299	0.0619	0.0160	0.067*	
C14	0.7539 (3)	0.08485 (8)	0.08053 (11)	0.0499 (4)	
C15	0.5403 (3)	−0.03522 (7)	0.22942 (10)	0.0495 (4)	
C16	0.7057 (3)	−0.06426 (9)	0.27877 (12)	0.0620 (5)	
H16	0.8411	−0.0447	0.2868	0.074*	
C17	0.6716 (3)	−0.12205 (9)	0.31627 (12)	0.0659 (5)	
H17	0.7840	−0.1404	0.3504	0.079*	
C18	0.4750 (3)	−0.15320 (8)	0.30446 (11)	0.0586 (5)	
C19	0.3114 (3)	−0.12401 (9)	0.25490 (12)	0.0614 (5)	
H19	0.1773	−0.1441	0.2457	0.074*	
C20	0.3415 (3)	−0.06556 (8)	0.21842 (11)	0.0572 (4)	
H20	0.2271	−0.0466	0.1863	0.069*	
C21	0.4416 (4)	−0.21754 (9)	0.34221 (15)	0.0851 (6)	
H21A	0.3101	−0.2353	0.3161	0.128*	
H21B	0.4281	−0.2151	0.4065	0.128*	
H21C	0.5654	−0.2432	0.3281	0.128*	
C22	0.2735 (3)	0.08010 (8)	0.27948 (11)	0.0509 (4)	
C23	0.3194 (3)	0.05472 (9)	0.36372 (12)	0.0622 (5)	
H23	0.4479	0.0320	0.3730	0.075*	
C24	0.1768 (3)	0.06271 (9)	0.43408 (12)	0.0671 (5)	
H24	0.2127	0.0459	0.4902	0.080*	
C25	−0.0174 (3)	0.09511 (9)	0.42289 (13)	0.0638 (5)	
C26	−0.0646 (3)	0.11931 (9)	0.33862 (13)	0.0641 (5)	
H26	−0.1963	0.1405	0.3289	0.077*	
C27	0.0785 (3)	0.11292 (8)	0.26840 (12)	0.0571 (4)	
H27	0.0439	0.1309	0.2128	0.068*	
C28	−0.1697 (3)	0.10576 (11)	0.50077 (14)	0.0894 (7)	
H28A	−0.1592	0.1483	0.5200	0.134*	
H28B	−0.1282	0.0789	0.5499	0.134*	
H28C	−0.3183	0.0969	0.4818	0.134*	

### Atomic displacement parameters (Å^2^)

**Table:**

	*U*^11^	*U*^22^	*U*^33^	*U*^12^	*U*^13^	*U*^23^
N1	0.0563 (9)	0.0479 (9)	0.0578 (8)	0.0029 (7)	0.0064 (7)	−0.0033 (7)
C2	0.0513 (9)	0.0470 (10)	0.0504 (9)	0.0003 (8)	0.0000 (8)	−0.0053 (7)
C3	0.0481 (9)	0.0491 (10)	0.0516 (9)	−0.0002 (8)	0.0015 (7)	−0.0062 (8)
N4	0.0543 (8)	0.0508 (9)	0.0603 (8)	0.0030 (7)	0.0080 (7)	−0.0010 (7)
C5	0.0501 (10)	0.0492 (10)	0.0548 (10)	0.0015 (8)	0.0030 (8)	−0.0033 (8)
C6	0.0592 (11)	0.0513 (11)	0.0682 (11)	0.0062 (9)	0.0067 (9)	0.0008 (9)
C7	0.0580 (11)	0.0516 (11)	0.0552 (10)	−0.0047 (8)	0.0011 (8)	−0.0020 (8)
C8	0.0749 (13)	0.0567 (12)	0.0736 (12)	−0.0015 (10)	0.0065 (10)	0.0068 (10)
C9	0.0868 (15)	0.0592 (13)	0.0727 (13)	−0.0142 (11)	0.0065 (11)	0.0076 (10)
C10	0.0758 (14)	0.0766 (14)	0.0666 (12)	−0.0194 (12)	0.0157 (10)	−0.0011 (11)
C11	0.0625 (12)	0.0691 (13)	0.0628 (11)	−0.0042 (10)	0.0099 (9)	−0.0067 (10)
C12	0.0566 (10)	0.0543 (11)	0.0480 (9)	−0.0059 (8)	0.0029 (8)	−0.0070 (8)
C13	0.0570 (10)	0.0534 (10)	0.0573 (10)	0.0060 (8)	0.0055 (8)	−0.0058 (8)
C14	0.0528 (10)	0.0468 (10)	0.0501 (9)	−0.0015 (8)	0.0031 (7)	−0.0053 (8)
C15	0.0517 (10)	0.0472 (10)	0.0496 (9)	0.0032 (8)	0.0039 (7)	−0.0053 (8)
C16	0.0535 (11)	0.0614 (12)	0.0709 (12)	0.0014 (9)	−0.0013 (9)	−0.0010 (10)
C17	0.0686 (13)	0.0642 (12)	0.0647 (11)	0.0130 (10)	−0.0037 (9)	0.0066 (10)
C18	0.0726 (13)	0.0516 (11)	0.0519 (10)	0.0035 (9)	0.0108 (9)	−0.0012 (8)
C19	0.0624 (12)	0.0572 (11)	0.0646 (11)	−0.0114 (9)	0.0025 (9)	−0.0047 (9)
C20	0.0581 (11)	0.0534 (11)	0.0597 (10)	−0.0003 (9)	−0.0061 (8)	−0.0009 (8)
C21	0.1156 (18)	0.0598 (13)	0.0806 (14)	0.0034 (12)	0.0196 (13)	0.0129 (11)
C22	0.0508 (10)	0.0469 (10)	0.0551 (10)	−0.0011 (8)	0.0045 (8)	−0.0082 (8)
C23	0.0620 (11)	0.0645 (12)	0.0604 (11)	0.0082 (9)	0.0059 (9)	−0.0029 (9)
C24	0.0761 (13)	0.0709 (13)	0.0546 (10)	0.0037 (11)	0.0111 (9)	−0.0033 (9)
C25	0.0612 (11)	0.0618 (12)	0.0690 (12)	−0.0062 (9)	0.0148 (9)	−0.0163 (10)
C26	0.0503 (10)	0.0659 (12)	0.0762 (13)	0.0038 (9)	0.0048 (9)	−0.0152 (10)
C27	0.0557 (10)	0.0563 (11)	0.0592 (10)	0.0002 (8)	0.0013 (8)	−0.0070 (8)
C28	0.0821 (15)	0.1055 (18)	0.0821 (14)	−0.0039 (13)	0.0310 (12)	−0.0222 (13)

### Geometric parameters (Å, º)

**Table:**

N1—C2	1.3102 (19)	C16—C17	1.380 (3)
N1—C14	1.381 (2)	C16—H16	0.9300
C2—C3	1.451 (2)	C17—C18	1.378 (2)
C2—C15	1.488 (2)	C17—H17	0.9300
C3—N4	1.310 (2)	C18—C19	1.376 (2)
C3—C22	1.491 (2)	C18—C21	1.509 (3)
N4—C5	1.379 (2)	C19—C20	1.383 (2)
C5—C6	1.383 (2)	C19—H19	0.9300
C5—C14	1.419 (2)	C20—H20	0.9300
C6—C7	1.391 (2)	C21—H21A	0.9600
C6—H6	0.9300	C21—H21B	0.9600
C7—C8	1.425 (2)	C21—H21C	0.9600
C7—C12	1.426 (2)	C22—C23	1.386 (2)
C8—C9	1.349 (2)	C22—C27	1.387 (2)
C8—H8	0.9300	C23—C24	1.382 (2)
C9—C10	1.400 (3)	C23—H23	0.9300
C9—H9	0.9300	C24—C25	1.378 (3)
C10—C11	1.357 (3)	C24—H24	0.9300
C10—H10	0.9300	C25—C26	1.378 (3)
C11—C12	1.427 (2)	C25—C28	1.514 (2)
C11—H11	0.9300	C26—C27	1.379 (2)
C12—C13	1.396 (2)	C26—H26	0.9300
C13—C14	1.385 (2)	C27—H27	0.9300
C13—H13	0.9300	C28—H28A	0.9600
C15—C20	1.380 (2)	C28—H28B	0.9600
C15—C16	1.380 (2)	C28—H28C	0.9600
			
C2—N1—C14	117.63 (14)	C15—C16—H16	119.7
N1—C2—C3	121.78 (15)	C18—C17—C16	121.64 (17)
N1—C2—C15	116.81 (14)	C18—C17—H17	119.2
C3—C2—C15	121.33 (14)	C16—C17—H17	119.2
N4—C3—C2	121.30 (14)	C19—C18—C17	117.44 (17)
N4—C3—C22	115.02 (14)	C19—C18—C21	121.03 (18)
C2—C3—C22	123.63 (15)	C17—C18—C21	121.51 (18)
C3—N4—C5	117.64 (14)	C18—C19—C20	121.59 (17)
N4—C5—C6	119.19 (15)	C18—C19—H19	119.2
N4—C5—C14	120.82 (15)	C20—C19—H19	119.2
C6—C5—C14	119.93 (15)	C15—C20—C19	120.46 (17)
C5—C6—C7	121.02 (16)	C15—C20—H20	119.8
C5—C6—H6	119.5	C19—C20—H20	119.8
C7—C6—H6	119.5	C18—C21—H21A	109.5
C6—C7—C8	122.04 (17)	C18—C21—H21B	109.5
C6—C7—C12	119.22 (16)	H21A—C21—H21B	109.5
C8—C7—C12	118.73 (16)	C18—C21—H21C	109.5
C9—C8—C7	120.99 (19)	H21A—C21—H21C	109.5
C9—C8—H8	119.5	H21B—C21—H21C	109.5
C7—C8—H8	119.5	C23—C22—C27	117.51 (15)
C8—C9—C10	120.29 (19)	C23—C22—C3	123.20 (15)
C8—C9—H9	119.9	C27—C22—C3	119.05 (15)
C10—C9—H9	119.9	C24—C23—C22	120.96 (17)
C11—C10—C9	121.31 (18)	C24—C23—H23	119.5
C11—C10—H10	119.3	C22—C23—H23	119.5
C9—C10—H10	119.3	C25—C24—C23	121.47 (18)
C10—C11—C12	120.39 (18)	C25—C24—H24	119.3
C10—C11—H11	119.8	C23—C24—H24	119.3
C12—C11—H11	119.8	C26—C25—C24	117.47 (16)
C13—C12—C7	119.21 (15)	C26—C25—C28	121.05 (19)
C13—C12—C11	122.58 (17)	C24—C25—C28	121.44 (19)
C7—C12—C11	118.21 (16)	C25—C26—C27	121.64 (17)
C14—C13—C12	121.16 (16)	C25—C26—H26	119.2
C14—C13—H13	119.4	C27—C26—H26	119.2
C12—C13—H13	119.4	C26—C27—C22	120.91 (17)
N1—C14—C13	120.54 (15)	C26—C27—H27	119.5
N1—C14—C5	120.22 (14)	C22—C27—H27	119.5
C13—C14—C5	119.24 (16)	C25—C28—H28A	109.5
C20—C15—C16	118.33 (16)	C25—C28—H28B	109.5
C20—C15—C2	120.02 (15)	H28A—C28—H28B	109.5
C16—C15—C2	121.62 (16)	C25—C28—H28C	109.5
C17—C16—C15	120.51 (17)	H28A—C28—H28C	109.5
C17—C16—H16	119.7	H28B—C28—H28C	109.5
			
C14—N1—C2—C3	−3.0 (2)	C6—C5—C14—N1	−175.17 (16)
C14—N1—C2—C15	173.87 (14)	N4—C5—C14—C13	−172.58 (15)
N1—C2—C3—N4	7.3 (2)	C6—C5—C14—C13	4.4 (2)
C15—C2—C3—N4	−169.37 (15)	N1—C2—C15—C20	−119.48 (17)
N1—C2—C3—C22	−169.84 (15)	C3—C2—C15—C20	57.4 (2)
C15—C2—C3—C22	13.5 (2)	N1—C2—C15—C16	58.5 (2)
C2—C3—N4—C5	−3.6 (2)	C3—C2—C15—C16	−124.69 (17)
C22—C3—N4—C5	173.74 (14)	C20—C15—C16—C17	−0.3 (3)
C3—N4—C5—C6	179.40 (16)	C2—C15—C16—C17	−178.24 (16)
C3—N4—C5—C14	−3.6 (2)	C15—C16—C17—C18	1.4 (3)
N4—C5—C6—C7	173.89 (16)	C16—C17—C18—C19	−1.1 (3)
C14—C5—C6—C7	−3.2 (3)	C16—C17—C18—C21	177.36 (17)
C5—C6—C7—C8	−179.68 (17)	C17—C18—C19—C20	−0.2 (3)
C5—C6—C7—C12	−1.0 (3)	C21—C18—C19—C20	−178.70 (17)
C6—C7—C8—C9	176.35 (18)	C16—C15—C20—C19	−1.0 (2)
C12—C7—C8—C9	−2.3 (3)	C2—C15—C20—C19	176.97 (15)
C7—C8—C9—C10	−0.3 (3)	C18—C19—C20—C15	1.3 (3)
C8—C9—C10—C11	2.3 (3)	N4—C3—C22—C23	−139.82 (17)
C9—C10—C11—C12	−1.7 (3)	C2—C3—C22—C23	37.5 (2)
C6—C7—C12—C13	4.0 (2)	N4—C3—C22—C27	34.4 (2)
C8—C7—C12—C13	−177.35 (16)	C2—C3—C22—C27	−148.26 (16)
C6—C7—C12—C11	−175.84 (16)	C27—C22—C23—C24	−0.9 (3)
C8—C7—C12—C11	2.9 (2)	C3—C22—C23—C24	173.46 (17)
C10—C11—C12—C13	179.29 (17)	C22—C23—C24—C25	1.2 (3)
C10—C11—C12—C7	−0.9 (3)	C23—C24—C25—C26	0.0 (3)
C7—C12—C13—C14	−2.7 (2)	C23—C24—C25—C28	−177.85 (18)
C11—C12—C13—C14	177.09 (16)	C24—C25—C26—C27	−1.6 (3)
C2—N1—C14—C13	176.18 (15)	C28—C25—C26—C27	176.32 (18)
C2—N1—C14—C5	−4.2 (2)	C25—C26—C27—C22	1.9 (3)
C12—C13—C14—N1	178.13 (15)	C23—C22—C27—C26	−0.7 (3)
C12—C13—C14—C5	−1.5 (2)	C3—C22—C27—C26	−175.22 (16)
N4—C5—C14—N1	7.8 (2)		

### Hydrogen-bond geometry (Å, º)

Cg1 and Cg2 are the centroids of the C7–C12 and the N1/C2/C3/N4/C5–C7/C12–C14 rings, respectively.

**Table:**

*D*—H···*A*	*D*—H	H···*A*	*D*···*A*	*D*—H···*A*
C19—H19···*Cg*1^i^	0.93	2.88	3.488 (3)	124
C27—H27···*Cg*2^ii^	0.93	2.91	3.601 (3)	132

Symmetry codes: (i) −*x*+1, −*y*, −*z*; (ii) *x*−1, *y*, *z*.
